# Supplementary material for: Validation of the family focused mental health practice questionnaire in measuring health and social care professionals’ family focused practice
Source: PLoS One. 2023 May 22;18(5):e0285835. doi: 10.1371/journal.pone.0285835 (PMC10202282; doi:10.1371/journal.pone.0285835)
Supplement: S1 Table — (DOCX) [file pone.0285835.s001.docx]

**Supporting Information**

**Supplementary Table 1.**

*Correlations Matrix of 14 Factor Solution*

|  |  | Factors | | | | | | | | | | | | |
| --- | --- | --- | --- | --- | --- | --- | --- | --- | --- | --- | --- | --- | --- | --- |
|  | 1 | 2 | 3 | 4 | 5 | 6 | 7 | 8 | 9 | 0 | 11 | 12 | 13 | 14 |
| Factor1 | 1 |  |  |  |  |  |  |  |  |  |  |  |  |  |
| Factor2 | 0.097 | 1 |  |  |  |  |  |  |  |  |  |  |  |  |
| Factor3 | -0.135***** | 0.175***** | 1 |  |  |  |  |  |  |  |  |  |  |  |
| Factor4 | -0.046 | 0.368***** | 0.117***** | 1 |  |  |  |  |  |  |  |  |  |  |
| Factor5 | 0.086 | 0.168***** | 0.201***** | 0.01 | 1 |  |  |  |  |  |  |  |  |  |
| Factor6 | -0.123***** | 0.044 | 0.131***** | 0.223***** | 0.063 | 1 |  |  |  |  |  |  |  |  |
| Factor7 | -0.133***** | 0.116***** | 0.263***** | 0.193***** | 0.195***** | 0.138***** | 1 |  |  |  |  |  |  |  |
| Factor8 | -0.091***** | 0.279***** | 0.177***** | 0.172***** | 0.292***** | -0.015 | 0.197***** | 1 |  |  |  |  |  |  |
| Factor9 | -0.024 | -0.016 | 0.027 | 0.138 | 0.025 | -0.07 | 0.043 | 0.058 | 1 |  |  |  |  |  |
| Factor10 | 0.132***** | 0.261***** | 0.144***** | 0.357***** | 0.108***** | 0.01 | 0.058 | 0.141***** | -0.007 | 1 |  |  |  |  |
| Factor11 | -0.110***** | 0.017 | 0.293***** | 0.031 | 0.212***** | 0.156***** | 0.077***** | 0.262***** | 0.077 | 0.035 | 1 |  |  |  |
| Factor12 | -0.268***** | 0.033 | 0.091 | 0.131 | -0.024 | 0.018 | 0.024 | 0.039 | -0.087 | 0.008 | 0.154***** | 1 |  |  |
| Factor13 | -0.281***** | -0.001 | 0.207***** | 0.146***** | -0.018 | 0.092 | 0.018 | -0.005 | -0.055 | 0.064 | 0.214***** | 0.185***** | 1 |  |
| Factor14 | -0.079 | 0.153***** | 0.155***** | 0.147***** | 0.093***** | 0.022 | 0.06 | 0.280***** | -0.118***** | 0.058 | 0.254***** | 0.093 | 0.078 | 1 |

*Correlations with* ******* *are significant at p < .05*
