# Supplementary material for: Validation of the family focused mental health practice questionnaire in measuring health and social care professionals’ family focused practice
Source: PLoS One. 2023 May 22;18(5):e0285835. doi: 10.1371/journal.pone.0285835 (PMC10202282; doi:10.1371/journal.pone.0285835)
Supplement: S2 Table — (DOCX) [file pone.0285835.s002.docx]

**Supplementary Table 2**

*Items Factor Loadings (Est.=Estimate), Standard Errors (S.E.) and z of Items by Factors in the 12-Factor Solution*

| Training | Est. | S.E. | z |  | Connectedness | Est. | S.E. | z |  | Workplace support | Est. | S.E. | z |
| --- | --- | --- | --- | --- | --- | --- | --- | --- | --- | --- | --- | --- | --- |
| ITEM 29 | 0.689 | 0.021 | 32.141 |  | ITEM 15 | 0.72 | 0.028 | 25.429 |  | ITEM 1 | 0.629 | 0.025 | 24.987 |
| ITEM 39 | 0.861 | 0.014 | 62.947 |  | ITEM 32 | 0.739 | 0.031 | 23.69 |  | ITEM 5R | 0.598 | 0.028 | 21.267 |
| ITEM 44 | 0.934 | 0.011 | 86.119 |  | ITEM 41R | 0.331 | 0.035 | 9.433 |  | ITEM 6 | 0.545 | 0.028 | 19.72 |
| ITEM 46 | 0.663 | 0.022 | 30.486 |  |  |  |  |  |  | ITEM 18R | 0.795 | 0.019 | 42.847 |
| ITEM 56 | 0.709 | 0.026 | 27.401 |  |  |  |  |  |  | ITEM 23 | 0.65 | 0.025 | 25.852 |
|  |  |  |  |  |  |  |  |  |  | ITEM 22R | 0.803 | 0.018 | 44.683 |
| Skill and Knowledge to support parents/parenting | Est. | S.E. | z |  | Referral | Est. | S.E. | z |  | Psycho education | Est. | S.E. | z |
| ITEM 49 | 0.722 | 0.021 | 34.957 |  | ITEM 33 | 0.651 | 0.035 | 18.621 |  | ITEM 24 | 0.696 | 0.035 | 19.827 |
| ITEM 50 | 0.788 | 0.017 | 47.212 |  | ITEM 37R | 0.629 | 0.034 | 18.559 |  | ITEM 36 | 0.713 | 0.034 | 21.198 |
| ITEM 51 | 0.813 | 0.016 | 52.277 |  | ITEM 43 | 0.544 | 0.034 | 16.038 |  | ITEM 47 | 0.262 | 0.047 | 5.572 |
| ITEM 52 | 0.756 | 0.019 | 39.041 |  |  |  |  |  |  |  |  |  |  |
| ITEM 53 | 0.655 | 0.024 | 27.317 |  |  |  |  |  |  |  |  |  |  |
| Understanding The Family Model | Est. | S.E. | z |  | Skills and knowledge to support children | Est. | S.E. | z |  | Child protection | Est. | S.E. | z |
| ITEM 54 | 0.991 | 0.022 | 45.554 |  | ITEM 11 | 0.46 | 0.033 | 13.848 |  | ITEM 57 | 0.563 | 0.037 | 15.168 |
| ITEM 55 | 0.706 | 0.025 | 28.753 |  | ITEM 30R | 0.77 | 0.029 | 26.953 |  | ITEM 64 | 0.594 | 0.037 | 16.034 |
| ITEM 65R | 0.712 | 0.023 | 30.54 |  | ITEM 40R | 0.737 | 0.026 | 28.369 |  | ITEM 66 | 0.843 | 0.041 | 20.346 |
|  |  |  |  |  | ITEM 41R | 0.516 | 0.034 | 15.076 |  |  |  |  |  |
|  |  |  |  |  | ITEM 48R | 0.56 | 0.033 | 17.183 |  |  |  |  |  |
| Time and workload | Est. | S.E. | z |  | Service availability | Est. | S.E. | z |  | Engagement issues | Est. | S.E. | z |
| ITEM 3R | 0.632 | 0.031 | 20.111 |  | ITEM 2R | 0.554 | 0.038 | 14.663 |  | ITEM 38R | 0.518 | 0.044 | 11.836 |
| ITEM 20R | 0.778 | 0.023 | 34.287 |  | ITEM 14R | 0.694 | 0.032 | 21.768 |  | ITEM 61R | 0.673 | 0.038 | 17.801 |
| ITEM 35 | 0.565 | 0.033 | 17.184 |  | ITEM 31R | 0.674 | 0.035 | 19.428 |  | ITEM 62R | 0.601 | 0.037 | 16.426 |
| ITEM 58R | 0.784 | 0.022 | 36.135 |  |  |  |  |  |  |  |  |  |  |
| ITEM 63R | 0.625 | 0.028 | 21.986 |  |  |  |  |  |  |  |  |  |  |
