# Supplementary material for: Validation of the family focused mental health practice questionnaire in measuring health and social care professionals’ family focused practice
Source: PLoS One. 2023 May 22;18(5):e0285835. doi: 10.1371/journal.pone.0285835 (PMC10202282; doi:10.1371/journal.pone.0285835)
Supplement: S3 Table — (DOCX) [file pone.0285835.s003.docx]

**Supplementary Table 3**

*Standardised Loadings, Standard Errors, and p Values of Item Loadings by Items and by Factors*

|  | Estimate | Std Error | p value |
| --- | --- | --- | --- |
| FACTOR #1 |  |  |  |
| I29 | 0.689 | 0.021 | <.001 |
| I39 | 0.861 | 0.014 | <.001 |
| I44 | 0.934 | 0.011 | <.001 |
| I46 | 0.663 | 0.022 | <.001 |
| I56 | 0.709 | 0.026 | <.001 |
| FACTOR #2 |  |  |  |
| I15 | 0.72 | 0.028 | <.001 |
| I32 | 0.739 | 0.031 | <.001 |
| I41R | 0.331 | 0.035 | <.001 |
| FACTOR #3 |  |  |  |
| I1 | 0.629 | 0.025 | <.001 |
| I5R | 0.598 | 0.028 | <.001 |
| I6 | 0.545 | 0.028 | <.001 |
| I18R | 0.795 | 0.019 | <.001 |
| I23 | 0.65 | 0.025 | <.001 |
| I22R | 0.803 | 0.018 | <.001 |
| FACTOR #4 |  |  |  |
| I49 | 0.722 | 0.021 | <.001 |
| I50 | 0.788 | 0.017 | <.001 |
| I51 | 0.813 | 0.016 | <.001 |
| I52 | 0.756 | 0.019 | <.001 |
| I53 | 0.655 | 0.024 | <.001 |
| FACTOR #5 |  |  |  |
| I33 | 0.651 | 0.035 | <.001 |
| I37R | 0.629 | 0.034 | <.001 |
| I43 | 0.544 | 0.034 | <.001 |
| FACTOR #6 |  |  |  |
| I24 | 0.696 | 0.035 | <.001 |
| I36 | 0.713 | 0.034 | <.001 |
| I47 | 0.262 | 0.047 | <.001 |
| FACTOR #7 |  |  |  |
| I54 | 0.991 | 0.022 | <.001 |
| I55 | 0.706 | 0.025 | <.001 |
| I65R | 0.712 | 0.023 | <.001 |
| FACTOR #8 |  |  |  |
| I11 | 0.46 | 0.033 | <.001 |
| I30R | 0.77 | 0.029 | <.001 |
| I40R | 0.737 | 0.026 | <.001 |
| I41R | 0.516 | 0.034 | <.001 |
| I48R | 0.56 | 0.033 | <.001 |
| FACTOR #9 |  |  |  |
| I57 | 0.563 | 0.037 | <.001 |
| I64 | 0.594 | 0.037 | <.001 |
| I66 | 0.843 | 0.041 | <.001 |
| FACTOR #10 |  |  |  |
| I3R | 0.632 | 0.031 | <.001 |
| I20R | 0.778 | 0.023 | <.001 |
| I35 | 0.565 | 0.033 | <.001 |
| I58R | 0.784 | 0.022 | <.001 |
| I63R | 0.625 | 0.028 | <.001 |
| FACTOR #11 |  |  |  |
| I2R | 0.554 | 0.038 | <.001 |
| I14R | 0.694 | 0.032 | <.001 |
| I31R | 0.674 | 0.035 | <.001 |
| FACTOR #12 |  |  |  |
| I38R | 0.518 | 0.044 | <.001 |
| I61R | 0.673 | 0.038 | <.001 |
| I62R | 0.601 | 0.037 | <.001 |
