# Supplementary material for: Validation of the family focused mental health practice questionnaire in measuring health and social care professionals’ family focused practice
Source: PLoS One. 2023 May 22;18(5):e0285835. doi: 10.1371/journal.pone.0285835 (PMC10202282; doi:10.1371/journal.pone.0285835)
Supplement: S1 File — (DOCX) [file pone.0285835.s004.docx]

## Supplementary Table 4

## *Family Focused Mental Health Practice Questionnaire (FFMHPQ)*

**
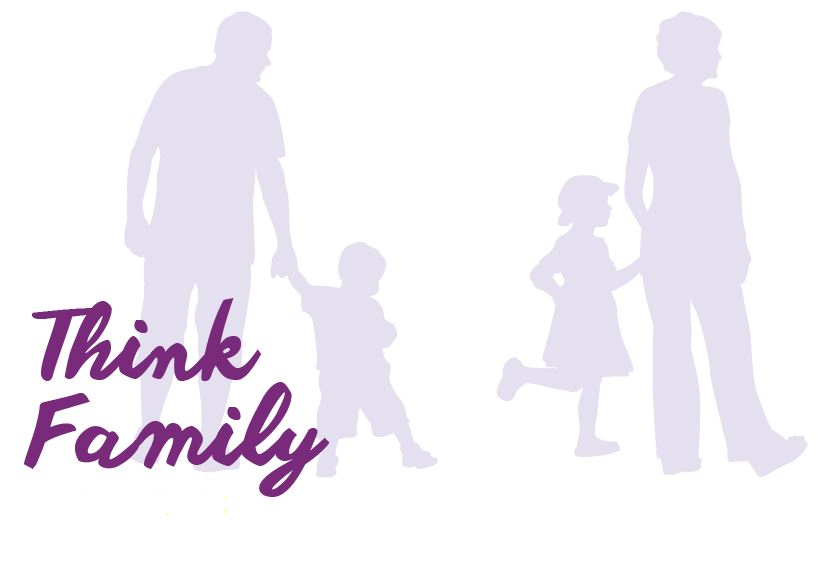
**

**Health and Social Care Professionals’ Family Focused Practice with Parents who have Mental Illness, their Children and Families in Northern Ireland**


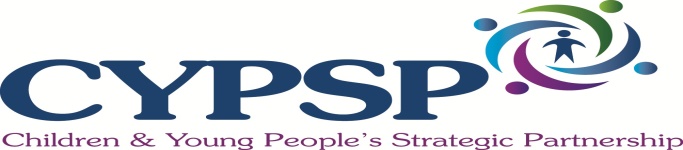


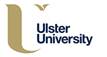

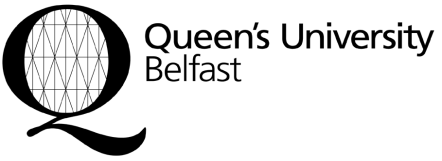

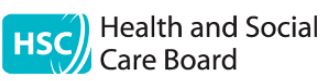


Since 2012 Think Family Northern Ireland (NI) has become core business for the Health and Social Care Board (HSCB) under the structure of the Children and Young People’s Strategic Partnership (CYPSP). This has resulted in a range of activities and projects designed to improve health and social care professionals’ understanding of family focused practice (FFP) and multidisciplinary working, while also improving the interface between services.

The aim of this survey is to gain an understanding of your practice with parents who have mental illness, their dependent children (18 or under) and other adult family members. The survey also explores your practice with other adult family members (including parents whose adult children have mental illness).

For the purpose of this study mental illness refers to a wide range of mental health conditions and disorders that affect mood, thinking and behaviour. Examples of mental illness include depression, anxiety disorders, schizophrenia, personality disorders and substance misuse.

The term **FFP is equal to Think Family**. Either term is generally used within this research to describe the process of working with parents who have mental illness around issues related to parenting with a mental illness, which may affect their parenting capacity and their own and their dependent children’s wellbeing. It also entails working with dependent children whose parents have mental illness to support them to cope with their parents’ mental illness. Your practice in relation to adult carers in general is also considered.

While adult mental health professionals are asked to consider their practice with parents who have mental illness using adult mental health services, social workers in children’s services should focus on their practice with parents who are receiving treatment for mental illness from either adult mental health services or primary care services, such as a GP. The survey can be completed even if you have no current or previous experience of caring for parents who have mental illness and/or for dependent children whose parent has/had mental illness. It is designed to rate your:

1. Perspective about statements relating to your knowledge and skill about family issues,

2. Interest in engaging in FFP to support children whose parent has mental illness, parents who have mental illness and families,

3. Perception of organisational policy and supports for FFP, and your level and type of FFP undertaken in your work, if any.

What is involved? This survey has three parts and will take up to 35 minutes to complete. There are no right or wrong answers; we are simply interested in obtaining feedback on health and social care professionals’ FFP and organisational support for FFP. The findings will generate understanding of health and social care professionals’ perspectives of FFP which can be used to shape future family focused initiatives in adult mental health and children’s services in line with feedback. This important study has been approved and supported by your Trust and where possible may be completed during working hours

We would like to emphasise that your involvement is VOLUNTARY and to maintain your ANONYMITY please do not put any identifying information on the survey. Data collected as part of the survey will be held for 10 years and may be used within a number research studies. Remember that your responses are anonymous and confidentiality will be protected.

**PART 1: Demographics**

**The purpose of this part of the survey is to establish background information about you and the position /role that you have within your organisation. Please answer all of the following questions.**

**1.1 Which of the following describes how you think of yourself?**

Tick one box only

| Female | [ ] |
| --- | --- |
| Male  In another way | [ ]  [ ] |

**1.2 What age are you? _______ (years)**

**1.3 How long have you been practicing as a qualified and registered health and social care**

**professional? (Please indicate in how many Weeks, Months or Years?)”**

(Please indicate either weeks, months or years) _______ (weeks)

_______ (months)

_______ (years)

**1.4 What is your professional discipline?**

Please tick all that apply

| Nurse | [ ] |  |  |
| --- | --- | --- | --- |
| Social Worker | [ ] |  |  |
| Psychiatrist | [ ] |  |  |
| Psychologist | [ ] |  |  |
| Allied Health Professional | [ ] |  |  |

Other (please specify) ____________________________

**1.5 Have you had any family-focused training?**

Tick one box only

| YES | [ ] |
| --- | --- |
| NO | [ ] |

If **yes** please detail when

(i.e. undergraduate and/or

post qualifying), type,

length of training & if accredited.

__________________________

__________________________

__________________________

__________________________

**1.6 Have you had any solely child-focused training?**

Tick one box only

| YES | [ ] |
| --- | --- |
| NO | [ ] |

If **yes** please detail when

(i.e. undergraduate and/or

post qualifying), type,

length of training & if accredited

__________________________

__________________________

__________________________

**1.7 Have you had any Think Family focused training?**

| YES | [ ] |
| --- | --- |
| NO | [ ] |

If **yes** please detail when

(i.e. undergraduate and/or

post qualifying), type,

length of training &

if accredited

__________________________

__________________________

__________________________

**1.8 If you work in Children’s Social Care Services have you received any training in relation to adult mental health?**

Tick one box only

| YES | [ ] |
| --- | --- |
| NO | [ ] |

If **yes** please detail when

(i.e. undergraduate and/or

post qualifying), type,

length of training &

if accredited

__________________________

__________________________

__________________________

**1.9 Which HSC Trust are you currently employed by?**

Tick one box only

| Belfast Health & Social Care Trust | [ ] | Southern Health & Social Care Trust | [ ] |
| --- | --- | --- | --- |
| South Eastern Health & Social Care Trust | [ ] | Northern Health & Social Care Trust | [ ] |
| Western Health & Social Care Trust | [ ] |  |  |

**1.10 In which service area are you currently working in?** Tick one box only

(Note: We understand that the terms used below to describe each of the services may not exactly fit with your Trust. We would ask you to select the service which best describes your area of work. If you cannot do this using the list below, please use the 'Other (Please specify)’ Option

| Acute In-Pt Mental Health and Addiction Services | [ ] | Community Mental Health Team | [ ] |
| --- | --- | --- | --- |
| Crisis Resolution Home Treatment | [ ] | Single Point of Access | [ ] |

| Community Addiction Team | [ ] | 16+ Team | [ ] |
| --- | --- | --- | --- |
| Family Intervention Team | [ ] | Gateway Service | [ ] |

Specialist mental health service (Please specify) __________________________

Other (Please specify) ________________________

**1.11 How long have you been working in your current position?**

(Please indicate either weeks, months or years) _______ (weeks)

______ (months)

_______ (years)

**1.12 Where is your service located? (Tick urban if your service is predominantly based in a city**

**setting)** Tick one box only

| Predominantly Rural Location | [  ] |
| --- | --- |
| Predominantly Urban Location  Urban & Rural Location | [  ]  [ ] |

**1.13 On what basis are you currently employed?**

Tick one box only

| Full-time | [ ] |
| --- | --- |
| Job Share | [ ] |
| Part – time (No job share) | [ ] |
| Other (Please specify) ____________ | |

**1.14** **How long on average would a service user be involved in your service?**

Tick one box only

| Less than a week | [ ] |  |
| --- | --- | --- |
| Between 1 – 4 weeks | [ ] |  |
| Up to six months | [ ] |  |
| More than six months | [ ] |  |

**1.15 Which Agenda for Change band are you currently employed at?**

(Please specify) ________________________

**1.16 If you are an adult mental health professional what rota are you currently working on?**

Tick one box only

| Day duty | [ ] |
| --- | --- |
| Night duty | [ ] |

Both rotas [ ]

N/ A [ ]

**1.17 Are you primarily involved in delivering direct care to service users?**

Tick one box only

| YES | [ ] |
| --- | --- |
| NO | [ ] |

If **yes,** please specify number of service users or cases you are responsible for ________ (number)

**1.18 Do you spend a percentage of your time each week delivering care within the service user’s home environment?**

Tick one box only

| YES | [ ] |
| --- | --- |
| NO | [ ] |

If **yes**, please specify percentage of time spent in the home environment__________ (per week)

**1.19 Are you or will you be acting as a CHAMPION resource for your team?** (A Champion has a formal role and responsibility for providing Think Family information, promoting joint working between services and identifying any obstacles to better cooperation).

Tick one box only

| \| YES \| [ ] \| \| --- \| --- \| \| NO \| [ ] \| |  |
| --- | --- | --- | --- | --- | --- |

**PART 2: Family Focused Mental Health Practice Questionnaire Subscales**

The following scale is a standardised tool designed to rate the extent to which you agree or disagree with statements regarding FFP. While the majority of items are related to FFP in relation to parents who have mental illness, their children and families, some items relate to FFP in relation to adult carers in general. Please refer to your experience in your current position and provide a response for every item regardless of whether you have current, previous or no experience of caring for parents who have mental illness or for children whose parent(s) have mental illness. If you have no experience of caring for parents who have mental illness and/or for their children, please tick the not applicable (N/A) option for those items that ask you to comment on your actual activities with parents who have mental illness, their children and families.

In responding to the questions below, please use the following scale which ranges from (1) strongly disagree to (7) strongly agree and includes a (N/A) not applicable category. For each question, please circle the answer (number) that best corresponds with your experience.

| \| **Not applicable** \| **Strongly**  **Disagree** \| **Disagree** \| **Slightly**  **Disagree** \| **Neither agree or disagree** \| **Slightly**  **Agree** \| **Agree** \| **Strongly**  **agree** \| \| --- \| --- \| --- \| --- \| --- \| --- \| --- \| --- \| \| **N/A** \| **1** \| **2** \| **3** \| **4** \| **5** \| **6** \| **7** \| | | | | | | | | | | |
| --- | --- | --- | --- | --- | --- | --- | --- | --- | --- | --- | --- | --- | --- | --- | --- | --- | --- | --- | --- | --- | --- | --- | --- | --- | --- | --- |
| 1 | My workplace provides mentoring to support health and social care professionals undertaking FFP | **N/A** | **1** | **2** | **3** | **4** | **5** | **6** | **7** | |
| 2 | In my area we lack services (e.g. other agencies) to refer children to in relation to their parent’s mental illness (i.e. programs for children) | **N/A** | **1** | **2** | **3** | **4** | **5** | **6** | **7** | |
| 3 | There is no time to work with children whose parent has mental illness or substance misuse around issues related to parental mental illness | **N/A** | **1** | **2** | **3** | **4** | **5** | **6** | **7** | |
| 4 | Government policy regarding FFP is very clear | **N/A** | **1** | **2** | **3** | **4** | **5** | **6** | **7** | |
| 5 | Professional development regarding FFP is not encouraged at my work place | **N/A** | **1** | **2** | **3** | **4** | **5** | **6** | **7** | |
| 6 | I often receive support from co-workers in regard to FFP | **N/A** | **1** | **2** | **3** | **4** | **5** | **6** | **7** | |
| 7 | I regularly have family meetings (not therapy) with parents who have mental illness and their children | **N/A** | **1** | **2** | **3** | **4** | **5** | **6** | **7** | |
| 8 | I am not confident working with mentally ill parents on their parenting skills | **N/A** | **1** | **2** | **3** | **4** | **5** | **6** | **7** | |
| 9 | I don’t provide information to the carer and/or family about the service user’s medication and/or treatment | **N/A** | **1** | **2** | **3** | **4** | **5** | **6** | **7** | |
| 10 | Many parents who have mental illness do not consider their illness to be a problem for their children | **N/A** | **1** | **2** | **3** | **4** | **5** | **6** | **7** | |
| 11 | I am able to determine the developmental progress of children whose parent(s) has mental illness | **N/A** | **1** | **2** | **3** | **4** | **5** | **6** | **7** | |
| 12 | I sometimes wish that I was better able to help parents discuss the impact of their mental illness on their children | **N/A** | **1** | **2** | **3** | **4** | **5** | **6** | **7** | |
| 13 | I am knowledgeable about how parental mental illness impacts on children. | **N/A** | **1** | **2** | **3** | **4** | **5** | **6** | **7** | |
| 14 | There are no parent-related programs (e.g. parenting skills) to refer parents with mental illness to | **N/A** | **1** | **2** | **3** | **4** | **5** | **6** | **7** | |
| 15 | I am able to determine the level of importance that parents who have mental illness place on their children maintaining attendance at day to day activities such as school and hobbies (e.g. sport, dance) | **N/A** | **1** | **2** | **3** | **4** | **5** | **6** | **7** | |
| 16 | I do not refer children whose parent has mental illness to child focused (e.g. peer support) programs (other than child and adolescent mental health) | **N/A** | **1** | **2** | **3** | **4** | **5** | **6** | **7** | |
| 17 | Working with other health and social care professionals enhances my FFP | **N/A** | **1** | **2** | **3** | **4** | **5** | **6** | **7** | |
| 18 | My workplace does not provide mentoring to support health and social care professionals undertaking FFP | **N/A** | **1** | **2** | **3** | **4** | **5** | **6** | **7** | |
| 19 | Due to location it is difficult to coordinate families and children with the required services | **N/A** | **1** | **2** | **3** | **4** | **5** | **6** | **7** | |
| 20 | My workload is too high to do family focused work | **N/A** | **1** | **2** | **3** | **4** | **5** | **6** | **7** | |
| 21 | At my workplace, policies and procedures for working with parents who have mental illness on family issues are very clear | **N/A** | **1** | **2** | **3** | **4** | **5** | **6** | **7** | |
| 22 | My workplace provides little support for further training in FFP | **N/A** | **1** | **2** | **3** | **4** | **5** | **6** | **7** | |
| 23 | In my workplace other workers encourage FFP | **N/A** | **1** | **2** | **3** | **4** | **5** | **6** | **7** | |
| 24 | I provide written material (e.g. Think Family educational resources, leaflets) about parenting to parents who have mental illness | **N/A** | **1** | **2** | **3** | **4** | **5** | **6** | **7** | |
| 25 | I am not confident working with families of service user’s | **N/A** | **1** | **2** | **3** | **4** | **5** | **6** | **7** | |
| 26 | Rarely do I advocate for the carers and/or family when communicating with other professionals regarding the service users’ mental illness | **N/A** | **1** | **2** | **3** | **4** | **5** | **6** | **7** | |
| 27 | Discussing issues for the service user with others (including family) would breach their confidentiality | **N/A** | **1** | **2** | **3** | **4** | **5** | **6** | **7** | |
| 28 | I am able to assess the level of children’s involvement in their parent’s symptoms | **N/A** | **1** | **2** | **3** | **4** | **5** | **6** | **7** | |
| 29 | I should learn more about how to assist parents who have mental illness with their parenting | **N/A** | **1** | **2** | **3** | **4** | **5** | **6** | **7** | |
| 30 | I do not have the skills to work with parents who have mental illness about how parental mental illness impacts on children and families | **N/A** | **1** | **2** | **3** | **4** | **5** | **6** | **7** | |
| 31 | There are no family therapy or family counselling services to refer parents who have mental illness and their children to | **N/A** | **1** | **2** | **3** | **4** | **5** | **6** | **7** | |
| 32 | I am able to determine the level of importance that parents who have mental illness place on their children maintaining strong relationships with other family members (e.g. other parent, siblings) | **N/A** | **1** | **2** | **3** | **4** | **5** | **6** | **7** | |
| 33 | I refer parents who have mental illness to parent-related programs (e.g. parenting skills) | **N/A** | **1** | **2** | **3** | **4** | **5** | **6** | **7** | |
| 34 | Children and families ultimately benefit if health and social care professionals work together to solve the family’s problems | **N/A** | **1** | **2** | **3** | **4** | **5** | **6** | **7** | |
| 35 | There is time to have regular contact with other agencies regarding parents, families or children (i.e. interface groups such as family support hubs) | **N/A** | **1** | **2** | **3** | **4** | **5** | **6** | **7** | |
| 36 | I regularly provide information (including written materials) about mental health issues to children whose parent(s) has mental illness | **N/A** | **1** | **2** | **3** | **4** | **5** | **6** | **7** | |
| 37 | Rarely do I consider if referral to peer support program (or similar) is required by children whose parent(s) has mental illness | **N/A** | **1** | **2** | **3** | **4** | **5** | **6** | **7** | |
| 38 | Children often do not want to engage with me about their  parent’s mental illness | **N/A** | **1** | **2** | **3** | **4** | **5** | **6** | **7** | |
| 39 | I would like to undertake future training to increase my skills and knowledge for working with children whose parent(s) has mental illness | **N/A** | **1** | **2** | **3** | **4** | **5** | **6** | **7** | |
| 40 | I am not experienced in working with child issues associated with parental mental illness | **N/A** | **1** | **2** | **3** | **4** | **5** | **6** | **7** | |
| 41 | I am not able to determine the level of importance that parents who have mental illness place on their children maintaining strong relationships with others outside the family (e.g. other children/peers, school) | **N/A** | **1** | **2** | **3** | **4** | **5** | **6** | **7** | |
| 42 | Team-working skills are essential for all health and social care professionals providing family-focused care | **N/A** | **1** | **2** | **3** | **4** | **5** | **6** | **7** | |
| 43 | I often consider if referral to parent support programme (or similar) is required by parents who have mental illness | **N/A** | **1** | **2** | **3** | **4** | **5** | **6** | **7** | |
| 44 | I would like to undertake training in future to increase my skills and knowledge about helping mentally ill parents with their parenting | **N/A** | **1** | **2** | **3** | **4** | **5** | **6** | **7** | |
| 45 | I am skilled in working with parents who have mental illness in relation to maintaining the wellbeing and resilience of their children | **N/A** | **1** | **2** | **3** | **4** | **5** | **6** | **7** | |
| 46 | I want to have a greater understanding of how to work within the multidisciplinary team to support children and families | **N/A** | **1** | **2** | **3** | **4** | **5** | **6** | **7** | |
| 47 | I provide education sessions for adult family members (e.g. about the illness, treatment) | **N/A** | **1** | **2** | **3** | **4** | **5** | **6** | **7** | |
| 48 | I am not confident working with children whose parent(s) has mental illness | **N/A** | **1** | **2** | **3** | **4** | **5** | **6** | **7** | |
| 49 | I am knowledgeable about the key things that parents who have mental illness could do to maintain the wellbeing (and resilience) of their children | **N/A** | **1** | **2** | **3** | **4** | **5** | **6** | **7** | |
| 50 | I am able to identify how parenthood can precipitate a parent’s mental illness | **N/A** | **1** | **2** | **3** | **4** | **5** | **6** | **7** | |
| 51 | I am able to identify how parenthood can influence a parent’s mental illness | **N/A** | **1** | **2** | **3** | **4** | **5** | **6** | **7** | |
| 52 | I assess the impact of the parenting role on the parent’s mental health | **N/A** | **1** | **2** | **3** | **4** | **5** | **6** | **7** | |
| 53 | I suggest practical strategies to facilitate parents who have mental illness to manage the dual demands of their parenting role and their mental illness or substance misuse | **N/A** | **1** | **2** | **3** | **4** | **5** | **6** | **7** | |
| 54 | I understand how to use Falkov’s Family Model to guide my FFP | **N/A** | **1** | **2** | **3** | **4** | **5** | **6** | **7** | |
| 55 | I perceive that Falkov’s Family Model can guide my FFP | **N/A** | **1** | **2** | **3** | **4** | **5** | **6** | **7** | |
| 56 | I would need to undertake future training to increase my skills and knowledge for using Falkov’s Family Model in practice | **N/A** | **1** | **2** | **3** | **4** | **5** | **6** | **7** | |
| 57 | The regional child protection procedures are clear about when I should be concerned that a parent’s mental illness is impacting negatively on a child | **N/A** | **1** | **2** | **3** | **4** | **5** | **6** | **7** | |
| 58 | There is no time to work with families | **N/A** | **1** | **2** | **3** | **4** | **5** | **6** | **7** | |
| 59 | I discuss the impact of family functioning, on children’s well-being, with the service user’s adult family members/carers | **N/A** | **1** | **2** | **3** | **4** | **5** | **6** | **7** | |
| 60 | I would classify my interaction with children whose parent has mental illness as planned, purposeful involvement with therapeutic intervention | **N/A** | **1** | **2** | **3** | **4** | **5** | **6** | **7** | |
| 61 | Parents generally do not want to engage with me about the impact of their mental illness on their children | **N/A** | **1** | **2** | **3** | **4** | **5** | **6** | **7** | |
| 62 | Discussing the impact of parental mental illness on children with parents who have mental illness would compromise rapport with them | **N/A** | **1** | **2** | **3** | **4** | **5** | **6** | **7** | |
| 63 | Insufficient numbers of health and social care professionals (i.e. nurse, social worker, clinical psychologist) in my service reduces worker’s capacity to address parenting issues | **N/A** | **1** | **2** | **3** | **4** | **5** | **6** | **7** | |
| 64 | I am clear about the thresholds for instigating a formal child in need (UNOCINI) assessment when a parent’s mental illness is impacting on their child | **N/A** | **1** | **2** | **3** | **4** | **5** | **6** | **7** | |
| 65 | I do not understand how to use Falkov’s Family Model to guide  my FFP | **N/A** | **1** | **2** | **3** | **4** | **5** | **6** | **7** | |
| 66 | I know what to do if I was concerned that a parent’s mental illness was having a significant negative effect on a child | **N/A** | **1** | **2** | **3** | **4** | **5** | **6** | **7** | |

**67. If you have chosen N/A to any of the previous questions in the Family Focused Mental Health Practice Subscales, can you tell us why?**

**There is some evidence to suggest that health and social care professionals who are, or have been, parents of dependent children (under 18) may feel more comfortable and/or able to address mentally ill parent’s parenting roles than those without experience of parenting (Grant, 2014; Korhonen et al., 2010). The following questions focus on how comfortable you are regarding parenting and children generally. If you do not have children please indicate N/A to questions 68 & 69**

| 68 | In general I am very happy with my parenting. | **N/A** | **1** | **2** | **3** | **4** | **5** | **6** | **7** |
| --- | --- | --- | --- | --- | --- | --- | --- | --- | --- |
| 69 | I have confidence in my parenting skills | **N/A** | **1** | **2** | **3** | **4** | **5** | **6** | **7** |
| 70 | I feel comfortable around other people’s children (e.g. friends, family) | **N/A** | **1** | **2** | **3** | **4** | **5** | **6** | **7** |

**71 Would you like to make any additional comments regarding your FFP and/or organisational support for FFP?**

Tick one box only

| YES | [ ] |
| --- | --- |
| NO | [ ] |

If **yes** please elaborate

_____________________________________________

_____________________________________________

_____________________________________________

_____________________________________________

**Please proceed to Part 3**

**PART 3: Professional experience of working with parents, who have mental illness,**

**their children and families and perspectives of Think Family initiatives**

The purpose of this part of the survey is to establish the extent of your professional exposure and experience in caring for parents who have mental illness, their children and adult family members. It also seeks your perspective of key Think Family initiatives. The term ‘dependent children’ is used to describe children under the age of 18. Please answer all of the following questions irrespective of whether you have professional experience of caring for parents who have illness, their children and/or adult family members. The majority of items can be completed irrespective of which setting you are practicing in, i.e. Adult Mental Health or Children's Services. Please respond according to the setting you are in.

**3. 1 Currently do you provide professional services to parents who have mental illness and/or their dependent children?** (Tick one box only)

| YES | [ ] (Please specify number you are  currently involved with) ____ |
| --- | --- |
| NO  Not Sure | [ ]  [ ] |

**3.2 Have you previous experience of providing professional services to parents who have mental illness and/or their dependent children?**

Tick one box only

| YES | [ ] |
| --- | --- |
| NO  Not Sure | [ ]  [ ] |

**3.3 In your current position how regularly do you provide services to parents who have mental illness or their dependent children?**

Tick one box only

| Daily | [ ] | Few times a Year | [ ] |
| --- | --- | --- | --- |
| Weekly | [ ] | Never | [ ] (**Go to 3.6**) |
| Monthly | [ ] |  |  |

**3.4 During the last week did you discuss issues related to parenting with parents who have mental illness?**

| Yes | [ ] (**Go to 3.6**) |
| --- | --- |
| No | [ ] (**Go to 3.5**) |

**3.5 What factors and/or circumstances(s), if**

**any, deterred you from discussing parenting?**

(Please specify)

_____________________________________________

_____________________________________________

_____________________________________________

_____________________________________________

**3.6 In the past week did you have face to face contact with a child(ren) whose parent has mental illness?**

Tick one box only

| Yes | [ ] |
| --- | --- |
| No | [ ] (**Go to 3.9**) |

**3.7 Did you discuss issues related to their parent’s mental illness?**

Tick one box only

| Yes | [ ] (**Go to 3.9**) |
| --- | --- |
| No | [ ] |

**3.8 What factors and/or circumstances(s), if**

**any, deterred you from discussing issues**

**associated with parental mental illness?**

**(**Please specify**)**

_____________________________________________

_____________________________________________

_____________________________________________

_____________________________________________

**3.9 Are you aware of the Regional Joint Adult &**

**Children’s Services Protocol?**

Tick one box only

| Yes | [ ] |
| --- | --- |
| No | [ ] (**Go to 3.13**) |

- 1. **Does the Regional Joint Adult & Children’s**

**Services Protocol enable your FFP?**

Tick one box only

| Yes | [ ] |
| --- | --- |
| No | [ ] (**Go to 3.12**) |

**3.11 How does the Regional Joint Adult &**

**Children’s Services Protocol**

**enable your FFP? (**Please specify and

go to 3.13**)**

_____________________________________________

_____________________________________________

_____________________________________________

- 1. **What suggestions would you make for**

**strengthening the Regional Adult &**

**Children’s Services Joint Protocol?**

_____________________________________________

_____________________________________________

_____________________________________________

_____________________________________________

**3.13 Does current screening and assessment documentation (including UNOCINI – Appendix 1) facilitate you to address parents and children’s needs in relation to parental mental illness?**

Tick one box only

| Yes | [ ] |
| --- | --- |
| No | [ ] (**Go to 3.15**) |

**3.14 How does screening and assessment**

**documentation facilitate your FFP?**

**(**Please specify and go to 3.16**)**

_____________________________________________

_____________________________________________

_____________________________________________

_____________________________________________

- 1. **What suggestions would you make**

**for screening and assessment**

**documentation to facilitate your FFP?**

_____________________________________________

_____________________________________________

_____________________________________________

_____________________________________________

**3.16 Are you aware of Falkov’s Family Model?**

Tick one box only

| Yes | [ ] |
| --- | --- |
| No | [ ] (**Go to 3.19**) |

If **yes** how did you learn

About the Family Model _____________________________________________

_____________________________________________

**3.17 Do you use Falkov’s Family Model in your work?**

Tick one box only

| Yes | [ ] |
| --- | --- |
| No | [ ] (**Go to 3.18**) |
|  |  |
|  |  |

If **yes** how do you use

the Family Model (please

specify by providing examples

and go to 3.19) _____________________________________________

_____________________________________________

_____________________________________________

**3.18 If you do not use Falkov’s Family Model**

**Can you tell us why?**

_____________________________________________

_____________________________________________

_____________________________________________

___________________________________________

**3.19 If working in Adult Mental Health Services do**

**you support adult family members including**

**parents who have adult children with mental illness?**

Tick one box only

| Yes | [ ] |
| --- | --- |
| No | [ ] (**Go to 3.20**) |
| **If you work in children’s services please Go to 3.21** |  |
|  |  |

If **yes** how do you support

adult family members (please

specify by providing examples

and go to 3.21) _____________________________________________

_____________________________________________

_____________________________________________

_____________________________________________

**3.20** **If working in Adult Mental Health Services**

**why do you not support**

**adult family members?**

_____________________________________________

_____________________________________________

_____________________________________________

_____________________________________________

**3.21 If you have current, or recent, experience (within the last 12 months) of caring for one or more parents who have mental illness and/ or substance misuse, or have experience of caring for their dependent children would you be willing to take part in a semi-structured interview in order to discuss your experiences further?**

Tick one box only

| Yes | [ ] (Please complete the Interview  Volunteer slip & forward  with your questionnaire) |
| --- | --- |
| No | [ ] |

**Have you any additional comments that you would like to make regarding the capacity of other Think Family initiatives to promote FFP (i.e. Champion Model, Think Family Practitioner) and/or how FFP could be further promoted? If yes, please elaborate in the box below.**

**Thank you for completing this questionnaire. Your contribution in this research is greatly appreciated. Please return your completed questionnaire online or by freepost in the envelope provided to:**

Dr Susan Lagdon

School of Nursing and Midwifery

97 Lisburn Rd

Belfast BT9 7BL, N. Ireland. **Email:** [ThinkFamilyNIStudy@Gmail.Com](mailto:ThinkFamilyNIStudy@Gmail.Com)
