# Supplementary material for: Validation of the family focused mental health practice questionnaire in measuring health and social care professionals’ family focused practice
Source: PLoS One. 2023 May 22;18(5):e0285835. doi: 10.1371/journal.pone.0285835 (PMC10202282; doi:10.1371/journal.pone.0285835)
Supplement: Attachment — Submitted filename: Response to reviewers.docx [file pone.0285835.s007.docx]

**Rebuttal Letter**

| **Editor and reviewer comments** | **Author’s response** |
| --- | --- |
| **Journal requirements:**   1. Does manuscript meet PLOS ONE's style requirements. 2. Please provide additional details regarding participant consent. In the ethics statement in the Methods and online submission information, please ensure that you have specified (1) whether consent was informed and (2) what type you obtained (for instance, written or verbal, and if verbal, how it was documented and witnessed). 3. We note that Table 2 may include questionnaire items that may have been previously published. The reproduction of previously published work has implications for the copyright that may apply to these publications. We would be grateful if you could clarify whether you have obtained permission from the original copyright holder to republish these items under a CC BY license. 4. Please review your reference list to ensure that it is complete and correct. 5. The [PLOS Data policy](https://eur02.safelinks.protection.outlook.com/?url=http%3A%2F%2Fwww.plosone.org%2Fstatic%2Fpolicies.action%23sharing&data=04%7C01%7CA.Grant%40qub.ac.uk%7C79db0cd9966a4e43039608d9e5abf097%7Ceaab77eab4a549e3a1e8d6dd23a1f286%7C0%7C0%7C637793348900082502%7CUnknown%7CTWFpbGZsb3d8eyJWIjoiMC4wLjAwMDAiLCJQIjoiV2luMzIiLCJBTiI6Ik1haWwiLCJXVCI6Mn0%3D%7C3000&sdata=i1SezX%2BqjsMt6qLLr2JtxAVCGOTKwOAYFXi5F8AWaiE%3D&reserved=0) requires authors to make all data underlying the findings described in their manuscript fully available without restriction, with rare exception | Yes we have followed guidelines, including uploading figure to PACE.  Thanks for highlighting this lack of detail. On page 11 we have now included the following text: Participants gave informed consent; they were informed of the details of the study in online explanatory statements. Implied consent was obtained through participation in the completion of the online or hard copy, anonymous questionnaire.  We can confirm that we have obtained permission from the original copyright holder (Professor Darryl Mayberry/Monash University) to republish these items  We have reviewed our reference list and it is complete and correct.  We had informed participants of this study that data would be used within publications and presentations. However, while we are in support of the open science framework and are also committed to ethical data sharing we did not include any detail regarding data repositories within participant information. |
| **Reviewer 2:**   1. The manuscript mentioned that the FFMHPQ was refined within the Irish context (page 9) but what are the changes made were not discussed appropriately. 2. The reason for choosing the sites was also not discussed. What was the inclusion and exclusion criteria of selecting HSC professionals needs to elaborate. 3. Generalizability of the study needs to discusses both; at the national and global level. | Thankyou for your comment. On page 9 we had previously included the text below. We have now also included additional text in yellow below into page 9 as the primary change included development of 3 additional subscales. If this is not sufficient can the reviewer be more specific about what additional details they would like regarding adaption and we will provide?  As the FFMHPQ was devised for use in the Australian context, with a variety of professional disciplines (e.g. psychologists, psychiatric nurses, social workers), it required minor adaption and testing in the NI context particularly for HSC professionals practicing within adult mental health and children’s services. Accordingly, the term ‘consumer parent’ was changed to ‘service user’ (8). Further, in conjunction with the developers of the instrument in response to the emerging literature on PMI, FFP and organisational developments in FFP in NI, three additional subscales (containing 10 additional items) were included within the current survey. These new subscales (see Table 3 [factor 4,7 & 9]) aimed to measure professionals’ understanding of The Family Model and child protection protocols, and their interventions to reduce the impact of the parenting role on parental mental health.  Thanks for highlighting the need for the following information. On page 10 and 11 we have now included the following text:  We sought to include a wide range of HSC Professionals working across adult and children’s services with families where a parent has a mental illness. The survey respondents broadly mirror the relevant workforce which has been the focus of Think Family NI initiatives. The survey was distributed to Approx. 3585 HSC professionals within adult mental health and children’s services across the five HSC Trusts. The minimum number of HSC professionals needed to complete a survey (n = 878) was determined by various factors, including the size of the population to which results are generalizable to, the results of previous research and particularly findings from previous use of the FFMHPQ in different populations and countries and the overall purpose of the current study which was to compare two groups of HSC professionals with regard to their FFP. Hence, a two sample comparison of means was used to estimate the overall sample size.  We ensured that the characteristics of respondents reflected the population of HSC professionals who fulfilled the inclusion criteria (i.e. HSC professionals working within Adult Mental Health, Addictions services, Gateway, Family Interventions Teams (FIT) or 16+ Teams). To promote maximum variation and to secure sample access, a principal investigator (PI) for each Trust was identified along with and independent point of contact for the study. A total of 1088 survey questionnaires were accessed by HSC professionals giving a response rate of 30%. However, 119 of these were ineligible based on study inclusion/ exclusion criteria. Due to significant missing information, 101 cases were also removed from the dataset as more than 90% of the survey had not been completed and would not be suitable for inclusion in final analysis. The final sample comprised of 868 HSC professionals, a response rate of 24.2%. |
