# Supplementary material for: Validation of the family focused mental health practice questionnaire in measuring health and social care professionals’ family focused practice
Source: PLoS One. 2023 May 22;18(5):e0285835. doi: 10.1371/journal.pone.0285835 (PMC10202282; doi:10.1371/journal.pone.0285835)
Supplement: Attachment — Submitted filename: Response to reviewers & editor final.docx [file pone.0285835.s008.docx]

**Response Letter**

| **Editor and reviewer comments** | **Author’s response** |
| --- | --- |
| 1. Please review your reference list to ensure that it is complete and correct. 2. There are several language and grammar mistakes. Please proofread the documents. Some of the sentence use is not clear for example, “in the current study tow of the subscales, psychoeducation and child protection, had Cronbach’s alpha coefficients below 0.60, hence care should be taken when using these”. Please write complete sentences with clear indication. Please improve the language of manuscript. 3. Please add one heading on Theoretical Foundation of Family Focused Mental Health Practice Questionnaire. Please provide more details in table on how questions were developed. 4. Please include more literature review and background of the study. 5. Please add future recommendations and future directions of your study. | We have re-reviewed our reference list and it is complete and correct.  We have fully reviewed the manuscript and made some changes to help improve the language of the manuscript. Please see tracked changes throughout.  On page 6 we included a heading Theoretical Foundation of Family Focused Mental Health Practice Questionnaire and included detail of how initial items were generated in Australia by Maybery and Reupert. On page 10 we included detail regarding validity of the questionnaire.  Additional detail is included on page 6 regarding The Family Model - a framework that promotes family focused practice. We perceive that this further adds to the information regarding theoretical foundations of the FFMHPQ and detailed provided regarding additional subscales developed for NI context.  We have discussed our recommendations (including future direction for research) within our discussion section. For example, page 29 we state “Therefore, like existing research we feel it imperative for future research using the FFMHPQ in the context of multiple service/ sector assessment, to address and improve the reliability of the weaker items.  Page 30 “This connection between attitudes, knowledge and skill and behaviour highlights the importance of organisations having effective implementation strategies to embed FFP (43,34). Future research should further examine these relationships and identify key predictors of FFP within adult mental health and children’s services”  Page 30 “These differences suggest that while the measure is flexible it may have more relevance for some services, sectors and professionals than others, depending on the professionals’ remit, understanding and operalisation of FFP. This needs consideration in future research examining FFP in different services other than adult mental health and children’s services”  Page 31 “Our findings further underscore this distinction in knowledge, skills and practice between services and sectors and this should be factored into future benchmarks and organizational support of FFP.”  Additional detail has also been added into page 32 in relation to recommendations and future directions. |
